# The impact of social unrest due to systemic racism on underrepresented post-doctoral fellows and early-career faculty

**DOI:** 10.1017/cts.2022.445

**Published:** 2022-08-19

**Authors:** Gretchen E. White, Chelsea N. Proulx, Doris M. Rubio, Maya S. Thakar, Natalia E. Morone, Chantele Mitchell-Miland, Andrew D. Althouse, Audrey J. Murrell

**Affiliations:** 1 Institute for Clinical Research Education, University of Pittsburgh Schools of the Health Sciences, Pittsburgh, PA, USA; 2 General Internal Medicine, Boston University School of Medicine, Boston, MA, USA; 3 Boston Medical Center, Boston University, Boston, MA, USA; 4 University of Pittsburgh, School of Medicine, Pittsburgh, PA, USA; 5 College of Business Administration, University of Pittsburgh, Pittsburgh, PA, USA

**Keywords:** Social unrest, systemic racism, diversity equity inclusion, antiracism, career development

## Abstract

**Introduction:**

Social unrest tied to racism negatively impacted half of NIH-funded extramural researchers underrepresented (UR) in science. UR early-career scientists encounter more challenges in their research careers, but the impact of social unrest due to systemic racism in this group is unclear. We used mixed methods to describe the impact of social unrest due to systemic racism on mentoring relationships, research, and psychological well-being in UR post-doctoral fellows and early-career faculty.

**Methods:**

This is a cross-sectional analysis of data collected in September 2021–January 2022 from 144 UR early-career researchers from 25 academic medical centers in the Building Up Trial. The primary outcomes were agreement on five-point Likert scales with social unrest impact statements (e.g., “I experienced psychological distress due to events of social unrest regarding systemic racism”). Thematic analysis was conducted on responses to one open-ended question assessing how social unrest regarding systemic racism affected participants.

**Results:**

Most participants were female (80%), non-Hispanic Black (35%), or Hispanic (40%). Over half of participants (57%) experienced psychological distress as a result of social unrest due to systemic racism. Participants described direct and indirect discrimination and isolation from other persons of color at their institutions. Twice as many participants felt their mentoring relationships were positively (21%) versus negatively (11%) impacted by social unrest due to systemic racism.

**Conclusions:**

Experiences with racial bias and discrimination impact the career and well-being of UR early-career researchers. Mentoring relationships and institutional support play an important role in buffering the negative impact of racial injustice for this population.

## Introduction

Early-career scientists from underrepresented (UR) backgrounds encounter more challenges than those who are well represented as they progress through biomedical research careers [1] and they disproportionately leave their careers [2–4]. In 2020, a Racial Justice Movement was reawakened in the United States as the country watched George Floyd be murdered by Minneapolis police officers. People of color relived racial trauma when faced with social unrest related to the public murders of unarmed Black men and women during a time when the COVID-19 pandemic disproportionately impacted communities of color [6]. Qualitative research has illustrated the unique pain of UR individuals dealing with racial trauma related to social unrest and the COVID-19 pandemic[7].

Many academic medical organizations released statements in response to concerns about systemic racism in the aftermath of George Floyd’s death and made incremental changes in policy [8]. Yet little research has examined the impact of social unrest due to systemic racism on early-career scientists from UR backgrounds. Research from the National Institutes of Health shows that civil unrest tied to racism negatively impacted approximately half of extramural researchers who identified as women, Black or African American, or Hispanic [9]. However, these results were not available by career stage despite early-career researchers facing more obstacles than later stage researchers [10]. Therefore, using cross-sectional data from the Building Up a Diverse Biomedical Research Workforce Trial (Building Up) [11], the primary aim of this study was to use a mixed-methods approach to describe the impact of social unrest due to systemic racism on mentoring relationships, research, and psychological well-being in post-doctoral fellows and early-career faculty UR in science [12]. The secondary aim is to examine these findings by gender, race/ethnicity, and highest degree achieved.

## Methods

### Design and Participants

All participants were enrolled in the Building Up Trial, a cluster-randomized trial designed to diversify the biomedical research workforce at 25 institutions [11]. Building Up includes 224 post-doctoral fellows or early-career faculty who are UR in health-related sciences (i.e., from racial or ethnic groups UR in health-related sciences, with disabilities, from disadvantaged backgrounds, or are women) [12]. Building Up has been previously described [11]. Briefly, the trial compares two interventions lasting 10 months and including four components (i.e., mentoring, monthly sessions, networking, and coursework). This report includes participants from both intervention arms. A single Institutional Review Board at the University of Pittsburgh approved the protocol. Participants provided electronic informed consent and were informed that their responses were confidential.

Data for this manuscript are from the second annual assessment for Building Up, which included six questions on the impact of social unrest due to systemic racism. Data were collected from September 2021 to January 2022.

### Measures

Participants answered a series of six questions on the personal and professional impact of social unrest due to systemic racism. Questions are summarized in Supplemental Table 1. The five quantitative questions were collected concurrently with the one open-ended qualitative question.

### Data Analysis

An exploratory mixed methods convergent design using quantitative and qualitative approaches was used to understand the personal and professional impact of social unrest due to systemic racism on UR early-career biomedical researchers. The research team conducted separate analyses of quantitative and qualitative data in parallel.

SAS version 9.4 (SAS Institute, Cary, NC, USA) was used for quantitative analyses. Reported p-values are two-tailed; p-values <0.05 were deemed statistically significant. As this was predominantly an exploratory cross-sectional analysis, we did not account for multiple comparisons [13]. Participant characteristics and impacts of social unrest due to systemic racism are reported as medians and 25^th^ and 75^th^ percentiles for continuous data and frequencies and percentages for categorical data. To assess for potential selection bias, we compared participants included in versus excluded from the analysis sample using the Pearson Chi-square test for categorical variables and the Wilcoxon Rank Sum test for continuous variables. Differences in the impact of social unrest due to systemic racism by gender, race and ethnicity, and highest degree were also tested with the Chi-square test for categorical variables and the Wilcoxon Rank Sum test for continuous variables. Due to small sample sizes, the categories for social unrest due to systemic racism variables were collapsed (i.e., negatively or very negatively; no impact; positively or very positively) when examining these variables by race and ethnicity.

We conducted a thematic qualitative analysis [14,15] of responses to the open-ended question on how the social unrest regarding systemic racism affected participants’ professional/academic life in the past year. The primary coder (CNP) inductively [16] developed a draft codebook. The codebook was reviewed by the secondary coder (MST) for clarity and comprehensiveness of definitions, before the primary and secondary coders applied the codebooks to each participant response. The average Cohen’s Kappa, which assesses interrater reliability, was calculated and showed substantial agreement (Kappa = 0.71) [17]. The primary and secondary coder adjudicated disagreements until full agreement was reached for each response before performing thematic analysis. Potential differences by gender and race and ethnicity were explored.

## Results

**Fig. 1.**
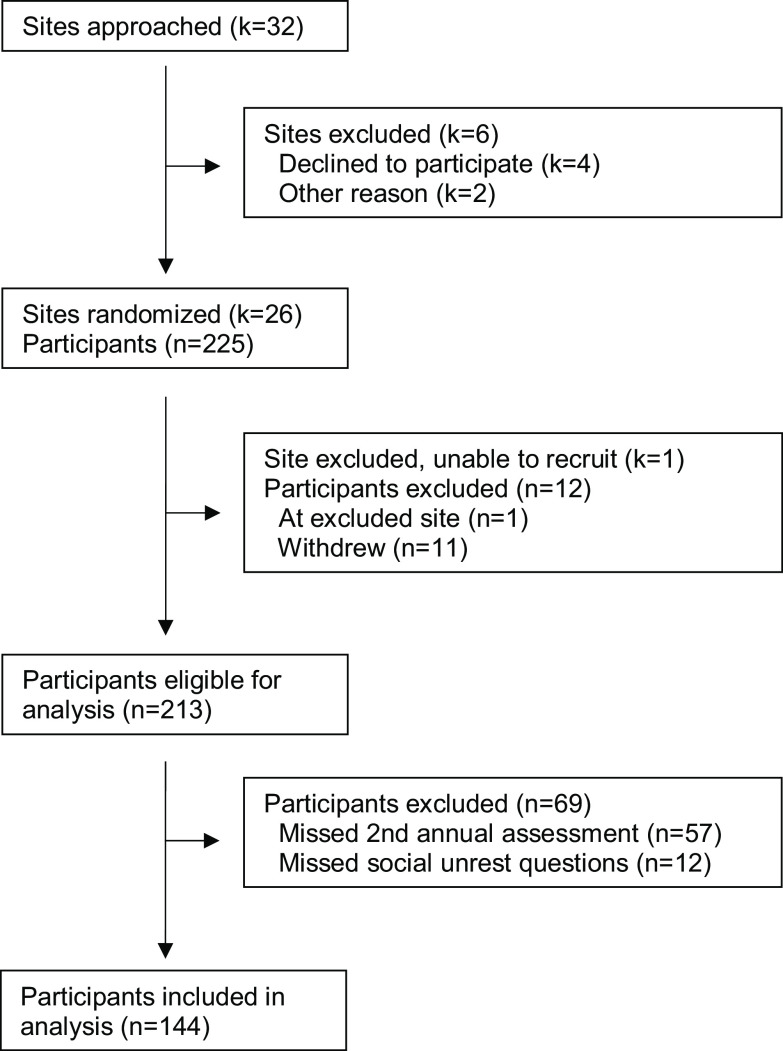
Institution and participant flow diagram for the building up a diverse biomedical research workforce trial.

Sixty-eight percent (144/213) of eligible participants completed at least one social unrest due to systemic racism question on the second annual assessment and were included in analyses (Fig. 1). Characteristics of participants included in and excluded from analyses are described in Table 1. For example, participants included in the analysis sample were significantly more likely to identify as Hispanic/Latinx or non-Hispanic/Latinx Black and less likely to identify as non-Hispanic/Latinx White than those excluded. The sample is 80% female, 40% Hispanic/Latinx, 35% non-Hispanic/Latinx Black, and 56% have a PhD. The median age was 36 years (25^th^–75^th^ percentile: 33–40).

**Table 1.** Characteristics of underrepresented post-doctoral fellows and early-career faculty included in versus excluded from analysis sample

	Included (*N* = 144)	Excluded (*N* = 69)	*P*-value^b^
Characteristic	n^a^ (%)	n^a^ (%)	
Age (median, 25^th^–75^th^ percentile)	36 (33–40)	37 (32–41)	0.56
Gender			0.23
Male	29 (20.3)	10 (14.7)	
Female	114 (79.7)	57 (83.8)	
Gender minority	0 (0.0)	1 (1.5)	
Race/ethnicity			<0.001
Hispanic/Latinx	58 (40.3)	15 (22.1)	
Non-Hispanic/Latinx			
White	9 (6.3)	19 (27.9)	
Black	50 (34.7)	19 (27.9)	
Other	18 (12.5)	10 (14.7)	
Multi-race	9 (6.3)	5 (7.4)	
Have disability	9 (6.8)	2 (3.2)	0.31
Type of highest agree achieved			0.12
MD	52 (36.1)	14 (20.6)	
PhD	80 (55.7)	45 (66.2)	
Other	12 (8.3)	9 (13.2)	

a Unless otherwise specified. The number of participants across categories may not sum to the total due to missing data.

b Chi-square test.

### Quantitative Impact of Social Unrest due to Systemic Racism

Half of participants (50.4%) serve on diversity-related committees (Table 2). Approximately twice as many participants stated that their mentoring relationships were positively or very positively impacted by social unrest due to systemic racism (21.0%) when compared to those who said that their mentoring relationships were negatively or very negatively impacted (10.5%). A sizable minority of participants disagreed or strongly disagreed with the statements that social unrest due to systemic racism impacted their ability to work (40.6%) and conduct research (49.3%). Despite less than a third of participants agreeing or strongly agreeing that social unrest due to systemic racism impacted their ability to work (29.3%) or conduct research (15.5%) over half (57.0%) felt that social unrest due to systemic racism led to psychological distress.

**Table 2.** Impact of social unrest due to systemic racism on under-represented post-doctoral fellows and early-career faculty by gender

	Total (*n* = 144)	Male (*n* = 29)	Female (*n* = 114)	
	n^a^ (%)	n^a^ (%)	n^a^ (%)	*P*-value^b^
Serve on diversity, curriculum, or recruitment committees	71 (50.4)	11 (39.3)	59 (52.7)	0.20
Social unrest due to systemic racism				
Affected mentoring relationships				0.83
Very negatively	2 (1.4)	0 (0.0)	2 (1.8)	
Negatively	13 (9.1)	3 (10.3)	10 (8.9)	
No impact	98 (68.5)	21 (72.4)	76 (67.3)	
Positively	27 (18.9)	4 (13.8)	23 (20.4)	
Very positively	3 (2.1)	1 (3.5)	2 (1.8)	
Impacted ability to work				0.14
Strongly disagree	15 (10.5)	1 (3.6)	13 (11.4)	
Disagree	43 (30.1)	12 (42.9)	31 (27.2)	
Neither agree nor disagree	44 (30.8)	11 (39.3)	33 (29.0)	
Agree	36 (25.8)	4 (14.3)	32 (28.1)	
Strongly agree	5 (3.5)	0 (0.0)	5 (4.4)	
Impacted ability to conduct research				0.10
Strongly disagree	19 (13.4)	1 (3.6)	17 (15.0)	
Disagree	51 (35.9)	13 (46.4)	38 (33.6)	
Neither agree nor disagree	50 (35.2)	13 (46.4)	37 (32.7)	
Agree	18 (12.7)	1 (3.6)	17 (15.0)	
Strongly agree	4 (2.8)	0 (0.0)	4 (3.5)	
Led to psychological distress				0.41
Strongly disagree	17 (12.0)	2 (7.1)	14 (12.4)	
Disagree	20 (14.1)	7 (25)	13 (11.5)	
Neither agree nor disagree	24 (16.9)	5 (17.9)	19 (16.8)	
Agree	56 (39.4)	9 (32.1)	47 (41.6)	
Strongly agree	25 (17.6)	5 (17.9)	20 (17.7)	

a The number of participants across categories may not sum to the total due to missing data.

b Chi-square test.

There were no statistically significant differences in the impact of social unrest due to systemic racism on mentoring relationships, the ability to work, the ability to conduct research, or experience of psychological distress by gender, race, and ethnicity, or highest degree achieved (Tables 2-4).

**Table 3.** Impact of the social unrest due to systemic racism on underrepresented post-doctoral fellows and early-career faculty by race and ethnicity

	Hispanic (*n* = 58)	Non-Hispanic White (*n* = 9)	Non-Hispanic Black (*n* = 50)	Non-Hispanic Other (*n* = 18)	Non-Hispanic Multi-race (*n* = 9)	*P*-value^b^
	n^a^ (%)					
Serve on diversity, curriculum, or recruitment committees	34 (60.7)	4 (44.4)	22 (44.9)	6 (33.3)	5 (55.6)	0.26
Social unrest due to systemic racism						
Affected mentoring relationships						0.43
Negatively or very negatively	8 (13.8)	0 (0.0)	4 (8.2)	2 (11.1)	1 (11.1)	
No impact	34 (58.6)	6 (66.7)	37 (75.5)	15 (83.3)	6 (66.7)	
Positively or very positively	16 (27.6)	3 (33.3)	8 (16.3)	1 (5.6)	2 (22.2)	
Impacted ability to work						0.15
Disagree or strongly disagree	18 (31.6)	7 (77.8)	22 (44.0)	6 (33.3)	5 (55.6)	
Neither agree nor disagree	19 (33.3)	1 (11.1)	15 (30.0)	5 (27.8)	4 (44.4)	
Agree or strongly agree	20 (35.1)	1 (11.1)	13 (26.0)	7 (38.9)	0 (0.0)	
Impacted ability to conduct research						0.40
Disagree or strongly disagree	23 (40.4)	7 (77.8)	27 (54.0)	7 (41.2)	6 (66.7)	
Neither agree nor disagree	22 (38.6)	2 (22.2)	16 (32.0)	7 (41.2)	3 (33.3)	
Agree or strongly agree	12 (21.1)	0 (0.0)	7 (14.0)	3 (17.7)	0 (0.0)	
Led to psychological distress						0.26
Disagree or strongly disagree	12 (21.1)	3 (33.3)	15 (30.6)	3 (16.7)	4 (44.4)	
Neither agree nor disagree	15 (26.3)	1 (11.1)	6 (12.2)	1 (5.6)	1 (11.1)	
Agree or strongly agree	30 (52.6)	5 (55.6)	28 (57.1)	14 (77.8)	4 (44.4)	

a The number of participants across categories may not sum to the total due to missing data.

b Chi-square test.

**Table 4.** Impact of the social unrest due to systemic racism on underrepresented post-doctoral fellows and early-career faculty by highest degree

	MD (*n* = 52)	PhD (*n* = 80)	Other (*n* = 12)	
	n^a^ (%)			*P*-value^b^
Serve on diversity, curriculum, or recruitment committees	28 (53.9)	39 (50.7)	4 (33.3)	0.44
Social unrest due to systemic racism				
Affected mentoring relationships				0.84
Very negatively	1 (1.9)	1 (1.3)	0 (0)	
Negatively	5 (9.6)	6 (7.6)	2 (16.7)	
No impact	36 (69.2)	55 (69.6)	7 (58.3)	
Positively	10 (19.2)	14 (17.7)	3 (25.0)	
Very positively	0 (0)	3 (3.8)	0 (0)	
Impacted ability to work				0.80
Strongly disagree	5 (9.6)	9 (11.4)	1 (8.3)	
Disagree	16 (30.8)	21 (26.6)	6 (50.0)	
Neither agree nor disagree	16 (30.8)	26 (32.9)	2 (16.7)	
Agree	14 (26.9)	20 (25.3)	2 (16.7)	
Strongly agree	1 (1.9)	3 (3.8)	1 (8.3)	
Impacted ability to conduct research				0.20
Strongly disagree	6 (11.8)	12 (15.2)	1 (8.3)	
Disagree	20 (39.2)	23 (29.1)	8 (66.7)	
Neither agree nor disagree	20 (39.2)	29 (36.7)	1 (8.3)	
Agree	4 (7.8)	13 (16.5)	1 (8.3)	
Strongly agree	1 (1.9)	2 (2.5)	1 (8.3)	
Led to psychological distress				0.19
Strongly disagree	5 (9.6)	11 (14.1)	1 (8.3)	
Disagree	4 (7.7)	12 (15.4)	4 (33.3)	
Neither agree nor disagree	12 (23.1)	11 (14.1)	1 (8.3)	
Agree	25 (48.1)	28 (35.9)	3 (25.0)	
Strongly agree	6 (11.5)	16 (20.5)	3 (25.0)	

a The number of participants across categories may not sum to the total due to missing data.

b Chi-square test.

### Qualitative Impact of Social Unrest due to Systemic Racism

Forty individuals (28.7% of sample) responded to the open text question, “Is there anything else you want us to know about how the social unrest regarding systemic racism affected your professional/academic life in the past year?” Those who completed the questions had similar demographic characteristics as the entire sample (i.e., 85% female, 30% non-Hispanic Black, and 43% Hispanic). Two themes were identified.

**Theme 1. Building Up participants across racial and ethnic groups described experiencing stress from social unrest due to systemic racism, exacerbated by overt discrimination or isolation from other persons of color at their institution.**

Almost half of respondents described experiencing some form of psychological distress as a result of social unrest due to systemic racism. Participants described feeling stress, frustration, exhaustion, emotional, and unsafe. Some described their experience as “tough,” recounting that the stress “consumes your mental energy.” One participant remarked that they needed to see a therapist after having a “breakdown over systemic racism.” Some participants described social unrest as a collective experience as illustrated by the participant below:

“The tax and stress that faculty and academic professionals of color have experienced over the past two years cannot be overstated. We are still dealing with the effects of the increasing public displays of racism and resistance to social justice and change, and that has immeasurable impacts on our psyche and mental health.” (Black, Female)

Others described their psychological distress more personally through recalling specific experiences of overt interpersonal discrimination and racism. For example, one participant who identified as Hispanic noted that the principal investigator of their lab considered their “minority grant” not as meritorious as other grants “open to ‘everybody’” and that the participant may not be a good fit as a professor because they did not “seem like or act like a ‘proper scientist.’” As illustrated in the quote below, another participant expressed how racist statements affected their feeling of safety:

“It was shocking to hear people in positions of power around me express racist and intolerant views in their conversations with me - people I would not have expected and it made me feel unsafe with them.” (American Indian/Alaska Native/White, Female)

For several participants, finding a support system at their institution of those who understood their experiences related to systemic racism was difficult and contributed to psychological distress, as the below participant describes:

“My division and fellowship program has no minority fellows or attendings. As the only minority fellow, I felt the leadership in my program could not relate with the distress I was going through during the racial social unrest protests and I did not feel supported. This significantly negatively affected my mental health. It was also a realization that even though I am at a top institution with lots of opportunities, this might not be the best culture for me to stay at long term.” (Hispanic, Female)

It is worth noting that not all participants were personally impacted by social unrest surrounding systemic racism. Two individuals noted that they work in international, multicultural research settings where they did not perceive any influence of systemic racism on their professional/academic life. One participant noted that “it is obvious it [systemic racism] exists, but has not affected me personally nor my job,” while another wrote, “you are assuming systemic racism exists and impacts everyone.”

**Theme 2. Building Up participants find hope and resilience in increased visibility, support, and open dialogue around Diversity, Equity, and Inclusion (DEI) at their institutions, while some worry that institutions are not doing enough to address systemic racism.**

Many participants remarked that social unrest due to systemic racism has prompted increased visibility of DEI at their institutions. Several noted that their own work in social justice received more support. Others described how increased awareness surrounding systemic racism inspired their own personal growth, noting that they now felt empowered to express their thoughts and feelings and advocate for themselves, like the participant below:

“It [social unrest due to systemic racism] really brought to light a lot of the experiences I think professional minorities try to suppress in order to face each day in professional environments that are not conducive to our development. It also made me more honest with myself about what impacts me and how much I’m willing to tolerate. It taught me vocabulary (e.g. microaggressions, gaslighting) for some of the most painful experiences I’ve been through in academia. It encouraged my friends and social circle of other minority professionals to share their own difficult experiences and helped me understand that we’ve ALL been through exactly the same things, no matter what our workplace looks like. And it gave me permission to stop pretending I’m ok with how I’m used and abused (or just feel like it) on a daily basis -- now I’m more vocal about setting boundaries and calling out intolerable behaviour.” (Black, Female)

While there was clear optimism around the increased awareness and visibility of DEI in academic spaces, not every participant felt their institution was doing enough to address disparities or gaps. For example, one participant noted that they were worried about “deeper systemic issues going unchallenged.” Another commented that “there is a long road ahead” at their institution, and that efforts to hire more diverse tenure-track faculty have not yet come to fruition.

## Discussion

Our primary research aim was to better understand the impact of social unrest due to systemic racism on psychological and professional well-being of UR faculty and post-doctoral fellows who are early in their career. This is an important question given the well-documented negative impact of dual pandemics of racism and COVID-19 on women and faculty of color [18]. Our unique mixed-method research design provides valuable insight into the lived experiences of these UR faculty and post-doctoral fellows. Our findings from both qualitative and quantitative data show that social unrest due to systemic racism had a substantial negative impact on psychological well-being for these post-doctoral fellows and early-career faculty. Conversely, despite less than a third of participants agreeing or strongly agreeing that social unrest due to systemic racism impacted their ability to work (29%) or conduct research (16%) in quantitative results, many participants described work-environments riddled with overt discrimination and isolation from other persons of color in qualitative results. These findings highlight that while early-career researchers are able to sustain their work and research responsibilities, they are often doing so in work environments that are not inclusive, not supportive, and sometimes hostile.

Participants’ reports of experiencing psychological distress and work interruptions also provides evidence of the impact of what prior research identifies as the negative impact of ambient discrimination [19]. Ambient discrimination recognizes that those who witness discrimination, harassment, and social unrest related to their social or identity group membership can suffer negative outcomes that are comparable to that experienced by the direct targets of discriminatory actions. Our findings provide clear support for the negative impact of ambient discrimination among post-doctoral fellows and early-career faculty. Together, with the disruption of the COVID-19 pandemic, early-career participants experienced compounded psychological distress, social isolation, and work disruptions during the critical early stages of their career. Putting into place strategies and efforts to reduce the negative consequences of ambient discrimination is essential to support the ongoing career advancement and positive well-being for early-career women and faculty of color.

Our findings suggest that one strategy for providing support for the impact of both systemic racism and ambient discrimination is the presence of social support and particularly mentoring relationships. As shared by our participants, finding a support system is an invaluable resource but one that is not always available to them at their institutions. Having access to social support and specifically mentoring relationships has been shown to have a positive buffering effect especially among UR faculty and researchers [20]. This includes diverse types of mentoring relationships including peer-to-peer mentoring and various forms of group mentoring especially based on shared affinity (e.g., functional area, background) or common social identity (e.g., race, gender, gender identity, culture, and ethnicity) [21]. The current results provide additional support from previous research showing a positive buffering effect of mentoring for the negative impact of both direct and ambient discrimination [22]. Our current findings are a timely discussion of the need to better understand the unique experiences and challenges faced by post-doctoral fellows and early-career faculty as part of our ongoing efforts toward diversity, equity, and inclusion in academic medical centers.

### Limitations

First, our sample was obtained from a randomized trial designed to test an intervention for people from UR backgrounds, including women. While the sample is drawn from 25 academic medical centers, it is not representative of all early-career scientists. Second, the sample size is small compared to some other studies. However, our mixed-methods design makes up for this limitation through qualitative findings that provide depth to the quantitative findings. Third, social unrest due to systemic racism is ongoing, and this study only assesses the impacts at one time point. The perspectives of participants will likely change over time and should be reassessed at a later time. While the mix of qualitative and quantitative questions is a strength of the current research, the need for more targeted questions (e.g., individual interviews) would provide additional information to reflect the different ways in which people recognize and value efforts to enhance diversity, equity, and inclusion. The fact that our sample has a significant number of women faculty relative to men could impact our findings and should also be addressed by future research efforts. Furthermore, our findings that there were no significant differences in the impact of social unrest due to systemic racism by gender, race, or ethnicity may be because all participants in this study were UR in sciences. Finally, our survey questions assumed that there is systemic racism in academia [23–25]; an assumption that one participant called into question. Such responses highlight the need for institutional support and policy changes that reduce racial inequality and bias in academia. In addition to the call for institutional support systems for people with shared experiences related to systemic racism that was brought up by participants in this study, Dupree and Boykin [23] suggest that institutions design policies to hire people of color and work to retain UR faculty through funding academic coaches and through developing and maintaining mentoring programs including those that are peer-based. Most importantly, these institutional policies should be created together with the people they are intended to benefit.

## Conclusions

Attempts to address systematic racism are complex and ongoing. However, efforts to support diverse scholars and faculty must address the range of their lived experiences within the social and professional environments in which they work and live. Our research points to the impact of both direct and indirect experiences with racial bias and discrimination on the career and well-being of diverse faculty. The current work also points to the important role that mentoring relationships and institutional support plays in providing a buffer for the negative impact of racial injustice for UR postdoctoral fellows and early-career faculty. Having the unique perspective of post-doctoral fellows and early-career faculty UR in science provides a necessary voice to existing literature on the impact of social unrest due to systemic racism as an important contribution of the current and future research.

## Supplementary material

For supplementary material accompanying this paper visit https://doi.org/10.1017/cts.2022.445. click here to view supplementary material
