# Supplementary material for: The impact of social unrest due to systemic racism on underrepresented post-doctoral fellows and early-career faculty
Source: J Clin Transl Sci. 2022 Aug 19;6(1):e112. doi: 10.1017/cts.2022.445 (PMC9549581; doi:10.1017/cts.2022.445)
Supplement: Supplementary file 1 [file S2059866122004459sup001.docx]

**Supplemental Table 1.** Impact of Social Unrest due to Systemic Racism Survey Questions

| **Question/Prompt** | **Response Options** |
| --- | --- |
| How has the social unrest regarding systemic racism affected your mentoring relationships? | Very negatively, negatively, no impact, positively, very positively |
| The social unrest regarding systemic racism has impacted my ability to work. | Strongly disagree, disagree, neither agree nor disagree, agree, strongly agree |
| The social unrest regarding systemic racism has impacted my ability to conduct research. | Strongly disagree, disagree, neither agree nor disagree, agree, strongly agree |
| I experienced psychological distress due to events of social unrest regarding systemic racism. | Strongly disagree, disagree, neither agree nor disagree, agree, strongly agree |
| I serve on university or department committees that address diversity, curriculum, or recruitment. | Yes, no |
| Is there anything else you want us to know about how the social unrest regarding systemic racism affected your professional/academic life in the past year? | Open-ended |
